# Endolysin Regulation in Phage Mu Lysis

**DOI:** 10.1128/mbio.00813-22

**Published:** 2022-04-26

**Authors:** Jake S. Chamblee, Jolene Ramsey, Yi Chen, Lori T. Maddox, Curtis Ross, Kam H. To, Jesse L. Cahill, Ry Young

**Affiliations:** a Center for Phage Technology, Department of Biochemistry and Biophysics, Texas A&M University, College Station, Texas, USA; Massachusetts Institute of Technology

**Keywords:** bacteriophage lysis, bacteriophages, membranes

## Abstract

Bacteriophage Mu is a paradigm coliphage studied mainly because of its use of transposition for genome replication. However, in extensive nonsense mutant screens, only one lysis gene has been identified, the endolysin gp22. This is surprising because in Gram-negative hosts, lysis by *Caudovirales* phages has been shown to require proteins which disrupt all three layers of the cell envelope. Usually this involves a holin, an endolysin, and a spanin targeting the cytoplasmic membrane, peptidoglycan (PG), and outer membrane (OM), respectively, with the holin determining the timing of lysis initiation. Here, we demonstrate that gp22 is a signal-anchor-release (SAR) endolysin and identify gp23 and gp23.1 as two-component spanin subunits. However, we find that Mu lacks a holin and instead encodes a membrane-tethered cytoplasmic protein, gp25, which is required for the release of the SAR endolysin. Mutational analysis showed that this dependence on gp25 is conferred by lysine residues at positions 6 and 7 of the short cytoplasmic domain of gp22. gp25, which we designate as a releasin, also facilitates the release of SAR endolysins from other phages. Moreover, the entire length of gp25, including its N-terminal transmembrane domain, belongs to a protein family, DUF2730, found in many Mu-like phages, including those with cytoplasmic endolysins. These results are discussed in terms of models for the evolution and mechanism of releasin function and a rationale for Mu lysis without holin control.

## INTRODUCTION

For release of progeny virions, the *Caudovirales* use “multigene lysis” (MGL) systems which involve at least two proteins, the holin and the endolysin (1). The holin initiates lysis by forming holes in the cytoplasmic membrane, which allows the endolysin, a muralytic enzyme, to attack the peptidoglycan (PG). Other MGL lysis proteins have been identified, including spanins and disruptins to attack the outer membrane and antiholins to regulate the timing of holin function (2, 3). Among the paradigm phages used to study most of the fundamental processes of prokaryotic biology, phage Mu stands out because its lysis system has not been delineated (4, 5). Extensive amber-mutant hunts generating more than 500 conditional lethal alleles have allowed the identification of 28 essential genes (6, 7). However, only one, designated *lys*, was associated with a defect in lysis independent of DNA replication (4, 5). In 2002, Morgan et al. (8) sequenced and annotated the complete Mu genome and unambiguously identified *lys* as gene *22*. No other lysis genes were identified. The presence of a distinctive catalytic triad identified its product, gp22, as a member of the “true lysozyme” (muraminidase/glycosidase/glycosyl hydrolase [9]) type of endolysins.

Subsequent bioinformatic analyses pointed to gp22 as a SAR (signal-anchor-release) endolysin (10, 11). Unlike soluble endolysins, which are released to the periplasm through micron-scale holes formed by canonical holins, SAR endolysins are exported in an inactive form by the Sec translocon. Tethering to the inner membrane (IM) depends on the eponymous SAR domain, a special type of N-terminal transmembrane domain (TMD) thought to require the proton motive force (PMF) for retention in the bilayer (1). Crucially, the membrane-tethered form of the SAR endolysin is catalytically inactive. Normally, the release of the SAR domain occurs suddenly and quantitatively due to holin triggering, but it can also occur at a low rate spontaneously when a holin is absent (12). The result is lysis in both cases, although the spontaneous release pathway generally occurs later and over a broader time spectrum than the normal holin triggering time. Most phages that use a SAR endolysin also encode a pinholin, which triggers to form nanometer-scale holes in the membrane rather than forming the micron-scale lesions characteristic of canonical holins (13).

Here, we report an experimental analysis of the Mu lysis system. The results not only demonstrate the existence of a novel lysis pathway in Mu, but also establish Mu as the first known tailed phage to lack a holin. The evolutionary implications for this lysis strategy in double-stranded DNA (dsDNA) phages are discussed.

## RESULTS

### Lysis genes of phage Mu.

**FIG 1:**
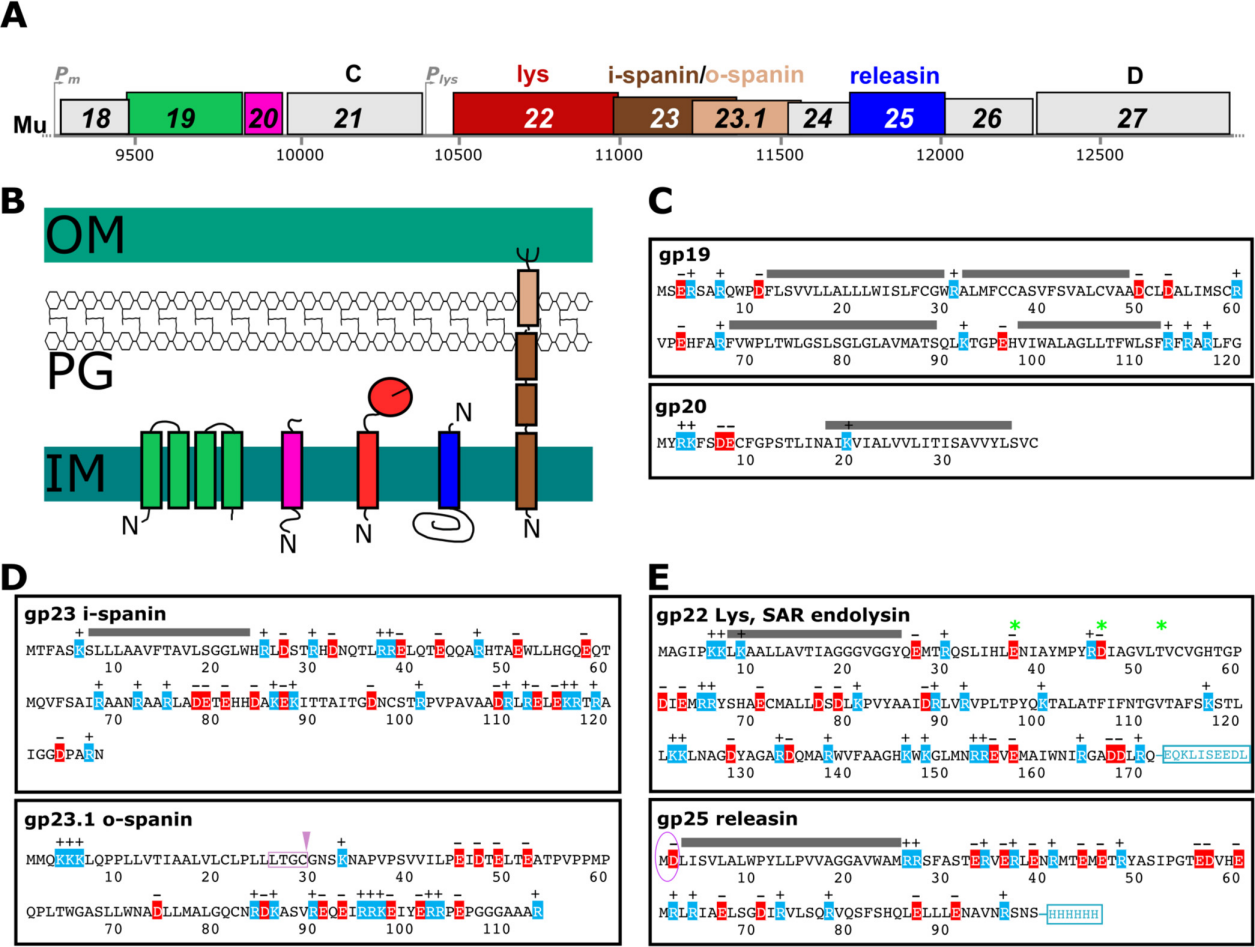
Mu membrane proteins and lysis genes. (A) Mu middle and late regions drawn to scale showing lysis genes in genomic context with nucleotide coordinates given below. The locations of middle- (P_m_) and late-transcription (P_lys_) promoters are indicated. Historic and new gene numbers are given inside the boxes. Functional assignments for lysis genes are listed above and predicted topologies are shown below in (B). (C to E) Primary structures of all identified Mu lysis proteins and transmembrane domain-containing proteins are shown in the boxes. Predicted transmembrane domains (TMDs) are shown as gray lines. Catalytic residues E-D-T in gp22 are marked with an asterisk. The Cys in the lipobox of the o-spanin is marked with an arrowhead. The added C-terminal epitope tags used in this study are shown in a blue box. Circled residues correspond to the predicted periplasmic domain of gp25.

**FIG 2:**
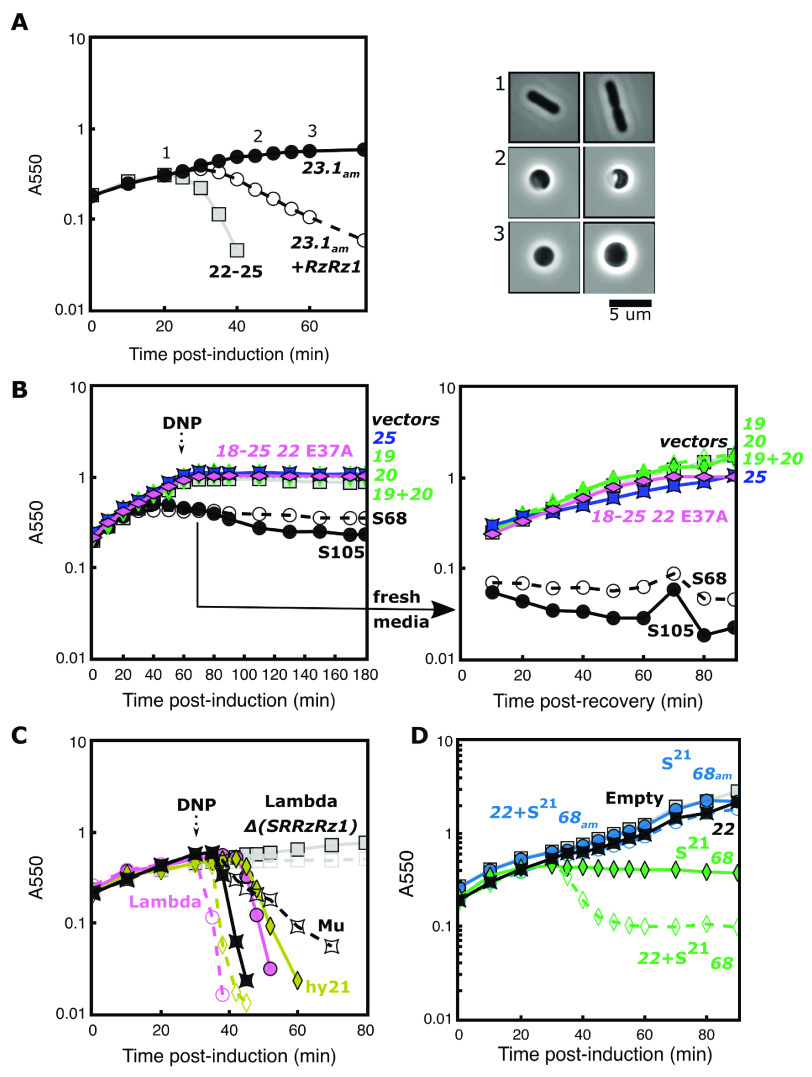
Mu lysis gene phenotypes. (A) Lysis genes in pBAD vectors were induced with arabinose in MG1655 cells grown in the presence of 10 mM Mg^2+^ in the following combinations: empty pBAD33 + pBAD18-Kan-Mu-gp22-gp25 (gray squares), empty pBAD33 + pBAD18-Kan-Mu-gp22-gp25-gp23.1*_am_* (“*23.1*_am_,” filled black circles), and λ spanins in pBAD33-λ-*RzRz1 *+* *pBAD18-Kan Mu-gp22-gp25-gp23.1*_am_* (“*23.1*_am_ + *RzRz1*,” open black circles). At 20, 45, and 60 min (marked 1, 2, and 3) *23.1_am_* samples were observed at 100× magnification for cellular morphology. (B) Mu lysis gene candidates cloned into pBAD vectors were induced with arabinose in MG1655 cells as follows: empty vectors (gray squares), pBAD24 gp25-his (*25*, blue star-squares), pBAD24 λ-*S105* (holins, black filled circle, solid line) and pBAD24 21-*S68* (holins, black open circle, dashed line), pBAD24-gp19 (open green diamond, dashed line), pBAD33-gp20 (open green diamond, dotted line), or together (*19 + 20*, filled green diamond, solid line). A 2-mM 2,4-dinitrophenol (DNP) treatment was applied at 60 min. A 5-mL aliquot of cells was pelleted at 70 min, then resuspended into fresh growth medium in new flasks to follow recovery in the absence of DNP. (C) MDS12 lysogens containing λ (pink circles), λ hy21 (mustard diamonds), λ Δ*(SRRzRz1)* (gray squares), or Mu*c^ts^* (black star-squares) were induced at 42°C. At 30 min, cells were treated with dimethyl sulfoxide (DMSO; solid shapes) or 1 mM DNP (open shapes, dashed line). (D) Lysis genes in pBAD vectors were induced with arabinose in MG1655 cells in the following combinations: empty pBAD33 and pBAD24 vectors (“empty,” gray squares), pBAD33-gp22-c-*myc* (“*22*,” filled black star square), pBAD24-21-S68 (“S^21^_68_,” filled green diamonds), pBAD24-21-S68 + pBAD33-gp22-c-*myc* (“*22* + S^21^_68_,” open green diamonds), pBAD24-21-S68_am_ (“S^21^_68am_,” filled blue circles), and pBAD24-21-S68_am_ + pBAD33-gp22-c-*myc* (“*22* + S^21^_68am_,” open blue circles).

In many phage genomes, the lysis genes are clustered (14). As noted above, the endolysin can be identified by the presence of one of the muralytic enzymatic domains, as in the case of gene *22* (*lys*). Generally, genes encoding the holin, antiholin and the two subunits of the spanin complex are nearby and co-transcribed. All four of these MGL proteins have transmembrane domains (TMDs) or membrane-targeting lipoprotein signals. At first inspection, Mu seems to fit this model. Indeed, in the Mu genome there are only four coding sequences (CDS) encoding proteins with at least one TMD (described in Methods), and all are near *22*: genes *19*, *20*, *23*, and *25* (Fig. 1 and Fig. S1 [in the supplemental material] for GenBank Accession no. AF083977). Gene *23* encodes a polypeptide with an N-terminal TMD and a periplasmic domain comprised of alpha-helical regions. We identified an overlapping CDS, *23.1*, not annotated in the original Mu genome, that encodes a short outer membrane (OM) lipoprotein rich in Pro residues (Fig. 1D). Indeed, amber mutations in these genes also demonstrate the classic spherical-cell phenotypes associated with the phage λ RzRz1 spanin defect in outer-membrane disruption (Fig. 2A) (15). Identification of the endolysin and spanin genes left only the holin and possible antiholin to complete the traditional lysis cluster. Of the three remaining membrane proteins, only gp25 is expressed from the late transcript, which is characteristic of all experimentally confirmed holins (16). Samanta et al. (10) proposed that gp19 and gp20 constituted the Mu holin-antiholin pair, based on predicted membrane topology matching other putative holins and antiholins. However, genes *19* and *20* are middle genes, rather than late genes. Moreover, previous genetic analysis had shown that both could be inactivated without a significant effect on lysis (17).

10.1128/mbio.00813-22.3 FIG S1 Mu genome annotation map. Annotations for the phage Mu*c^ts^* genome were assigned using the CPT Galaxy and Apollo systems (see Methods) and are reflected in the NCBI record for accession number AF083977. CDS, RBS, rho-independent terminators, and promoter features are shown. Download FIG S1, SVG file, 0.5 MB. Copyright © 2022 Chamblee et al. 2022 Chamblee et al. https://creativecommons.org/licenses/by/4.0/ This content is distributed under the terms of the Creative Commons Attribution 4.0 International license.

A definitive feature of holin function is that lethal premature triggering can be imposed by chemical depolarization of the membrane (16). This is true for both the canonical phage λ holin gene S105 and the phage 21 pinholin S68, which are devoid of their native antiholin/antipinholin regulatory components S107 and S71, respectively. To attempt to induce holin triggering, we cloned each of these holin and putative holin genes into an expression vector under arabinose control, grew the respective transformants until mid-logarithmic phase, induced with arabinose and then subjected the induced cultures to treatment with 2 mM 2,4-dinitrophenol (DNP) (18). Upon resuspension of the induced, DNP-treated cells in fresh medium, the cultures carrying the *19*, *20*, and *25* constructs showed unimpaired viability (Fig. 2B). In contrast, DNP-triggering of isogenic constructs with the well-characterized holin and pinholin genes of phage λ and phage 21 was lethal. Indeed, an additional isogenic construct carrying all the Mu holin candidates showed no lethality under the same conditions (this construct includes an inactivating E37A catalytic residue mutation in gp22, demonstrated to be inactive in Fig. 2B). These results suggest that Mu does not encode a holin. This conclusion is supported by DNP treatment with induced lysogens (Fig. 2C), where λ lysogens carrying either the canonical *S105* holin or the phage 21 pinholin both exhibit premature lysis upon addition of DNP. In contrast, DNP actually delays Mu lysis.

### Mu gp22 is a SAR endolysin.

**FIG 3:**
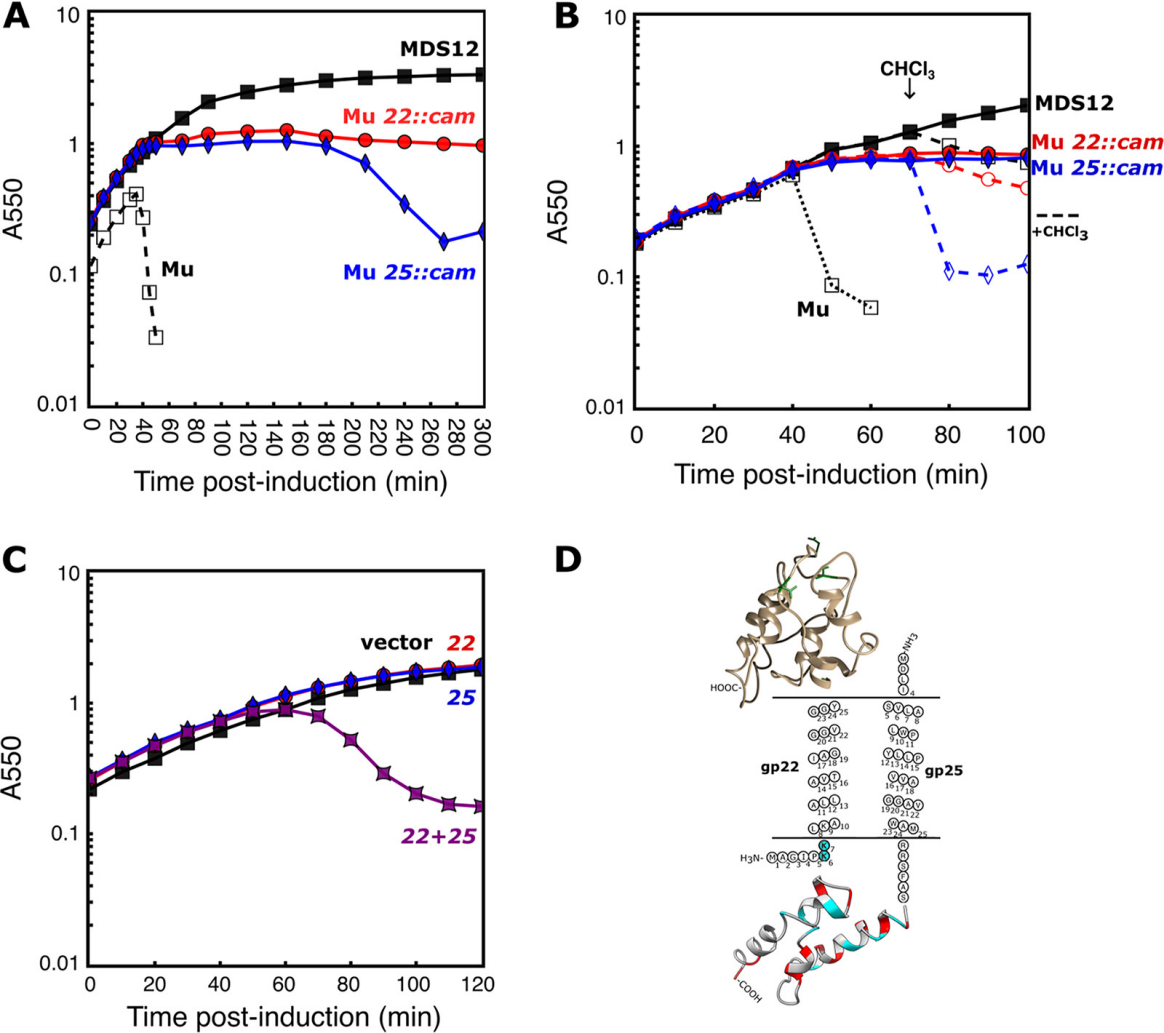
Gp22 relies on gp25 for release. (A) In MDS12 cells (filled squares) with a Mu*c^ts^* lysogen (open squares), Mu*c^ts^ 22::cam* (red circles) or Mu*c^ts^ 25*::*cam* (blue diamonds) are induced to replicate by a shift to 42°C at T = 0 min. (B) In the same experimental setup as used in panel A, 1% chloroform (dashed lines with open markers) was added to MDS12 (filled square), Mu*c^ts^ 22*::*cam* (red circle), and Mu*c^ts^ 25*::*cam* (blue diamond) at 70 min. (C) Mu lysis genes were cloned into pBAD vectors and induced in MG1655 cells as the following: empty vectors (black squares), pBAD24 gp25-his (*25*, blue diamonds) and pBAD33 gp22-c-*myc* (*22*, red circles) separately, or the latter two together (*22 *+* 25*, purple star squares). (D) Protein covariance calculated as significant intramolecular evolutionary coupling with EVcomplex was used as constraints for the gp22 catalytic domain and gp25 cytoplasmic domain folding. Catalytic residues of gp22 are shown in green as stick models. Charged residues in the predicted cytoplasmic regions are colored.

Previously, gp22 was recognized as the Mu endolysin because its product contains the canonical lysozyme catalytic triad motif E-X_8_-D-X_5_-T (19) (Fig. 3A). Closer inspection of the endolysin amino acid sequence revealed an N-terminal extension with the characteristics of a SAR domain: a weakly hydrophobic stretch of 20 residues beginning at position 6, of which 10 residues have little or no hydrophobic character (four Ala, five Gly, one Thr) (Fig. 1E). The definitive test for a SAR endolysin is whether it can be released from the IM by a pinholin. Fig. 2D demonstrates this for gp22 with the well-characterized phage 21 pinholin S^21^_68_.

### Mu gene 25 required for SAR endolysin release.

Our results indicated that none of the Mu membrane proteins had holin properties and that genes *22*, *23*, and *23.1* encoded the endolysin and spanin proteins. This raised the possibility that Mu relies on degradation of the PG following spontaneous activation of the SAR endolysin gp22 and disruption of the OM by the gp23/23.1 spanin complex. From this perspective, we wondered whether, despite its clustering with the other lysis genes and its predicted membrane localization, gp25 had no essential role in lysis. In the parental Mu lysogen, wild-type lysis occurs as a sharply defined event at ~40 min after thermal induction (Fig. 3A). Observation of individual induced lysogenic cells shows a classic slight rounding due to SAR endolysin activity prior to explosive cell lysis (Video S1) (15). However, when we compare Mu lysogens with deletions of *22* and *25* to the parental genotype, both deletion mutants continue to grow unabated (Fig. 3A). Eventually, at about 3 h after induction, bulk culture lysis is observed with the *25*::*cam* mutant but not the isogenic *22*::*cam* mutant. However, gp22 can be artificially released from the membrane with chloroform and function to effect lysis (Fig. 3B). The simplest interpretation of this is that gp25 is required for gp22 release and activation, at least until gp22 levels reach much higher levels those obtained during the normal vegetative cycle. To address this possibility, we constructed two plasmids encoding gp22 and gp25 with C-terminal c-*myc* and His-tags, respectively. When induced under conditions where spanin activity was not required (15), neither pBAD33-gp22-c-*myc* nor pBAD24-gp25-his alone caused lysis (Fig. 3C). In contrast, induction of cells carrying both plasmids led to lysis at ~50 min. Since gp25 is not required for gp22 maturation or activity, these results indicate that gp25 provides an ancillary function in SAR endolysin release which is not not currently provided by another known lysis protein, enabling the SAR endolysin gp22 to escape the IM. In view of this novel function, we designated gp25 as the Mu “releasin.”

10.1128/mbio.00813-22.2 Video S1 Montage of lysing E. coli MC4100 Mu*c^ts^* lysogenic cells at 50 minutes after prophage induction. Download Movie S1, AVI file, 2.0 MB. Copyright © 2022 Chamblee et al. 2022 Chamblee et al. https://creativecommons.org/licenses/by/4.0/ This content is distributed under the terms of the Creative Commons Attribution 4.0 International license.

### Basic residues in the cytoplasmic domain of gp22 confer dependence on gp25.

**FIG 4:**
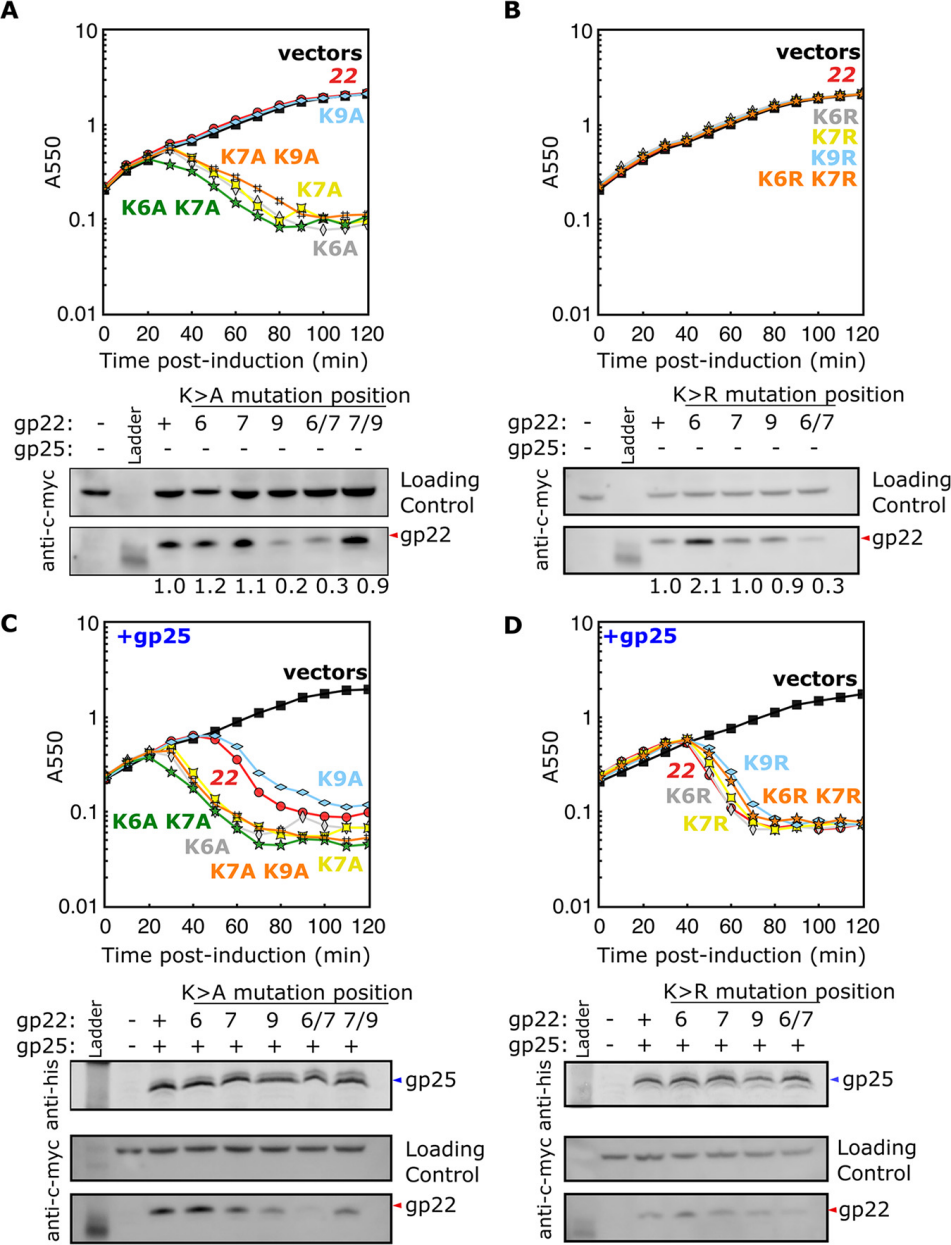
Cytoplasmic residue charge determines gp22 dependence on gp25. Gp22-c-*myc* alleles in pBAD33 with K→A (A and C) or K→R (B and D) mutations were assayed for lysis in MG1655 cells + 0.4% Ara. Gp22-c-*myc* alleles were assayed alone (A and B) and in the presence of pBAD24-gp25-his (C and D). Protein levels at 50 min post-induction are shown as detected by Western blotting.

gp25 is predicted to be a 99-aa (amino acid) type I (N-out, C-in) integral membrane protein with only four residues exposed in the periplasm and a 73-aa C-terminal cytoplasmic domain (Fig. 1E and 3D). The topology of gp22 is essentially the opposite, with the catalytic domain in the periplasm and only the short N-terminal extension in the cytoplasm. Most SAR endolysins have one or two N-terminal basic residues (12). Given our previous demonstration that adding basic residues at the cytoplasmic N terminus of a SAR domain can lock it in the bilayer (12), we hypothesized that the presence of basic residues, K6, K7, and K9, at the N terminus of gp22, confers dependence on gp25. To test this, we constructed plasmids carrying a panel of K→A substitution alleles of gp22-c-*myc*. First, the single Ala substitutions were tested for lytic function with or without the presence of gp25 (Fig. 4A and C). All three mutant gp22 alleles were found to be fully lytic with the co-expression of gp25 (Fig. 4C). Strikingly, both gp22*_K6A_* and gp22*_K7A_* also supported efficient lysis in the absence of gp25 (Fig. 4A). In contrast, gp22*_K9A_* alone retained the parental non-lytic phenotype. No significant increase in gp22 accumulation was detected for any of the missense changes; in fact, alleles with apparently slightly lower expression levels compared to the parental were still functional. Thus, it is the presence of lysine residues at both positions 6 and 7 that confers gp25 dependence on the escape of gp22 from the membrane. Moreover, the gp25 dependence was unchanged when either or both the lysine 6 and lysine 7 residues were substituted by arginines (Fig. 4B and D). These results indicate that it is the presence of positively charged residues at positions 6 and 7 that confers gp25 dependence for gp22 release.

### gp25 is a general “releasin” of SAR endolysins.

**FIG 5:**
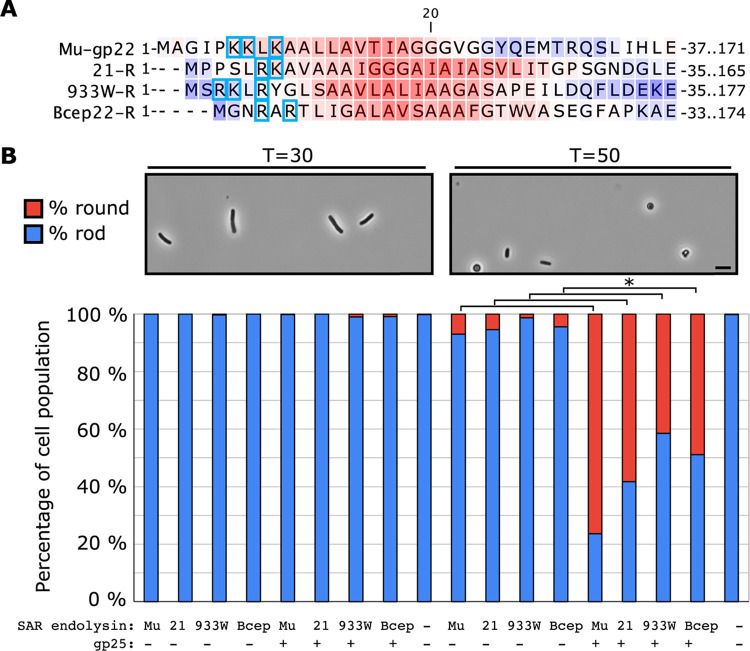
gp25 is a general releasin for SAR endolysins. (A) Alignment of the N-terminal regions of the four SAR endolysins, including the positively charged residues (boxed in blue) and the weakly hydrophobic residues of the SAR domain. All residue backgrounds are colored with the Kyte-Doolittle scale based on a 13-residue window in CLC Genomics Workbench. (B) SAR endolysins in pZE12 induced with isopropyl-β-D-thiogalactopyranoside (IPTG) at T = 40 were assayed with pBAD33 or pBAD33-gp25-his in MG1655 cells + 0.4% Ara at T = 0 min. Cell morphologies for ≥100 cells were observed before (at T = 30) and 10 min after endolysin induction (at T = 50). Scale bar is 5 μm. Example cell views are shown above the quantification averaged across three biological replicates. Samples with a significant difference by *t* test with *P* < 0.0001 are shown by an asterisk (*).

To investigate whether the mechanism of gp25 is specific to gp22, we cloned three other SAR endolysin genes from phages encoding holins and lacking genes resembling *25* into the isopropyl-β-d-thiogalactopyranoside (IPTG)-inducible pZE12 vector. Characteristic of SAR endolysins in general, all three have an N-terminal region with 2 to 3 basic residues followed by a TMD consisting of primarily weakly hydrophobic residues (Fig. 5A). Other than the presence of N-terminal basic residues, the SAR domains in the panel shared no apparent sequence patterns or motifs.

We then co-transformed each SAR endolysin plasmid with and without an arabinose-inducible pBAD33 vector encoding gp25. To prevent cell lysis after PG degradation, 10 mM Mg^2+^ was added to stabilize the outer membrane; under these conditions, lysis requires the presence of spanins. Consequently, if PG degradation occurs, cells are converted into spherical forms (15). At SAR endolysin expression levels achieved from the pZE vector, a small fraction of cells carrying only a SAR endolysin plasmid were converted to spherical forms (Fig. 5B). In contrast, a significantly higher proportion of cells co-expressing a SAR endolysin with gp25 were spherical, demonstrating that the ability of gp25 to facilitate SAR endolysin release is not specific to gp22.

### Bioinformatic analysis of endolysin-releasin pairs in phages.

**FIG 6:**
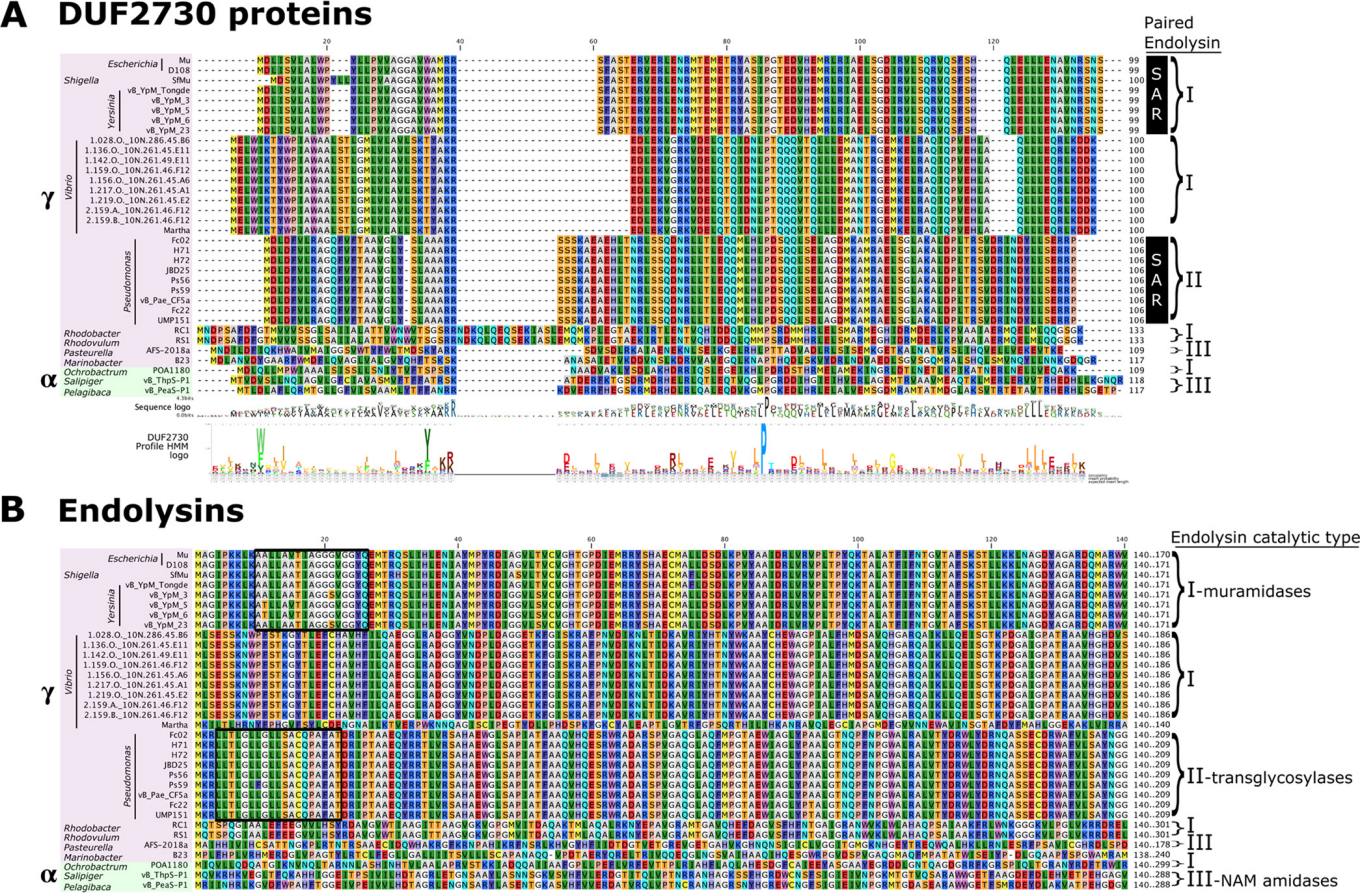
Alignment of SAR-endolysin-DUF2730 (releasin) pairs in phages. All active *Caudovirales* phages with the Mu gp25 DUF2730 domain found in InterPro were inspected for a paired endolysin. Protein sequences for (A) DUF2730 proteins and (B) endolysins were aligned and labeled according to phage and host (by genus) source, including host classification within the classes *Gammaproteobacteria* (γ, pink) or *Alphaproteobacteria* (α, green), and by endolysin catalytic type: I muramidase, II transglycosylase, and III NAM amidase, with letter designations for similarity groupings. SAR domains within the endolysins are boxed in black. The DUF2730 Profile HMM logo for the Pfam PF10805 domain is shown split similar to the alignment to illustrate collinearity (69, 70). Further information on phage, hosts, and conserved protein domains with accession numbers is detailed in Table S1 in the supplemental material.

Since there were few similarities shared among the SAR endolysins released by gp25, we wondered whether gp25-like proteins were genetically paired to endolysins with specific characteristics. SAR endolysins, however, are difficult to find by BLAST analysis since alignment results tend to favor similarity in the catalytic domain and localization signals are ignored as low-complexity features. Moreover, SAR domains are weakly hydrophobic and often are not recognized as TMDs by hydropathy-based algorithms. Therefore, we took advantage of the fact that the entire length of gp25, including the predicted N-terminal TMD, is defined by a conserved domain of unknown function, DUF2730. Thirty-four *Caudovirales* genomes carrying DUF2730 were found, all of which were Mu-like myophages or siphophages, with 31 gamma- and 3 alphaproteobacterial hosts (Fig. 6, Table S1). All the phages had a nearby endolysin gene, except *Vibrio* phage Martha 12B12, where synteny suggests that a soluble protein with weak similarity to L,d-transpeptidase, as assessed by HHPred, is the endolysin (20).

10.1128/mbio.00813-22.1 TABLE S1 Endolysin and releasin protein source information. A list of all endolysin and releasin protein pair accessions and their associated information: taxonomic lineage, number of genes in record and length of genes, source NCBI GenBank record, and host taxonomic classification. Download Table S1, XLSX file, 0.02 MB. Copyright © 2022 Chamblee et al. 2022 Chamblee et al. https://creativecommons.org/licenses/by/4.0/ This content is distributed under the terms of the Creative Commons Attribution 4.0 International license.

When each genome was inspected for the cognate endolysin gene, nine distinct sequence families could be discerned. Counting the Martha endolysin, four enzymatic classes of endolysin were represented, including four in the muramidase class, such as Mu gp22. Importantly, only two of the nine endolysin families had SAR domains, the Mu muramidase type and a transglycosylase type from Pseudomonas Mu-like siphophages. Thus, in diverse Mu-like phages, the DUF2730 proteins are paired with a great diversity of endolysins, with and without secretion signals.

## DISCUSSION

### Why does the gp22 SAR endolysin require another protein for release?

Our results show that the positively charged side chains at positions 6 and 7 confer gp25 dependence on the release and activation of gp22; replacement of either one with an Ala residue removes this dependency (Fig. 4). In contrast, the Lys residue at position 9 is irrelevant. Thus, this dependency is not due to the total positive charge in the cytoplasmic domain, which is presumably 4, counting the protonated N-terminal amino group and the three Lys residues. Instead, it is due to the arrangement of the charged residues. Unfortunately, nothing is known about the molecular details of SAR release. In the two cases where structural information was available, the details of the packing of the SAR domain into the globular periplasmic domain were vastly different (12, 21). Nevertheless, it is clear that extensive refolding of the endolysin occurs. The energetics and/or the kinetic pathway for these refolding events may influence the rate and extent of extraction of the SAR domain from the membrane. It is also possible that host factors interact with and influence the release of SAR endolysins. This certainly is true for the first SAR endolysin characterized, P1 Lyz, where the SAR domain undergoes covalent modification by the periplasmic Dsb system (12). SAR domains have not been systematically studied, so it is unclear what determines the relative stability in the bilayer, except that small, weakly hydrophobic residues are found at significantly higher rates in SAR domains compared to those in standard TMDs (12, 22, 23). One difference between the SAR domain of gp22 and other SAR endolysins found in late-transcript lysis cassettes carrying holin genes is that there is a stretch of seven positions containing six Gly residues (11, 12). No other SAR domain that has been characterized has more than two Gly residues. It is possible that the forces exerted from the periplasm which favor spontaneous release of the SAR domain may be decreased or dissipated by two full turns of easily extended Gly residues. In this regard, it is worth noting that the Mu SAR endolysin has a longer predicted cytoplasmic domain than other well-studied phage lysis systems (11).

### How does the gp25 releasin work?

This is the first example of a SAR endolysin which is dependent on a non-holin phage-encoded protein. The membrane topologies of the two proteins place conceptual constraints on models for gp25 function. gp25 consists of a predominantly anionic cytoplasmic domain attached to the membrane with an N-terminal TMD and has virtually no periplasmic component, whereas gp22 has the classic SAR endolysin topology, with its enzymatic domain in the periplasm and only a short N-terminal domain in the cytoplasm.

**FIG 7:**
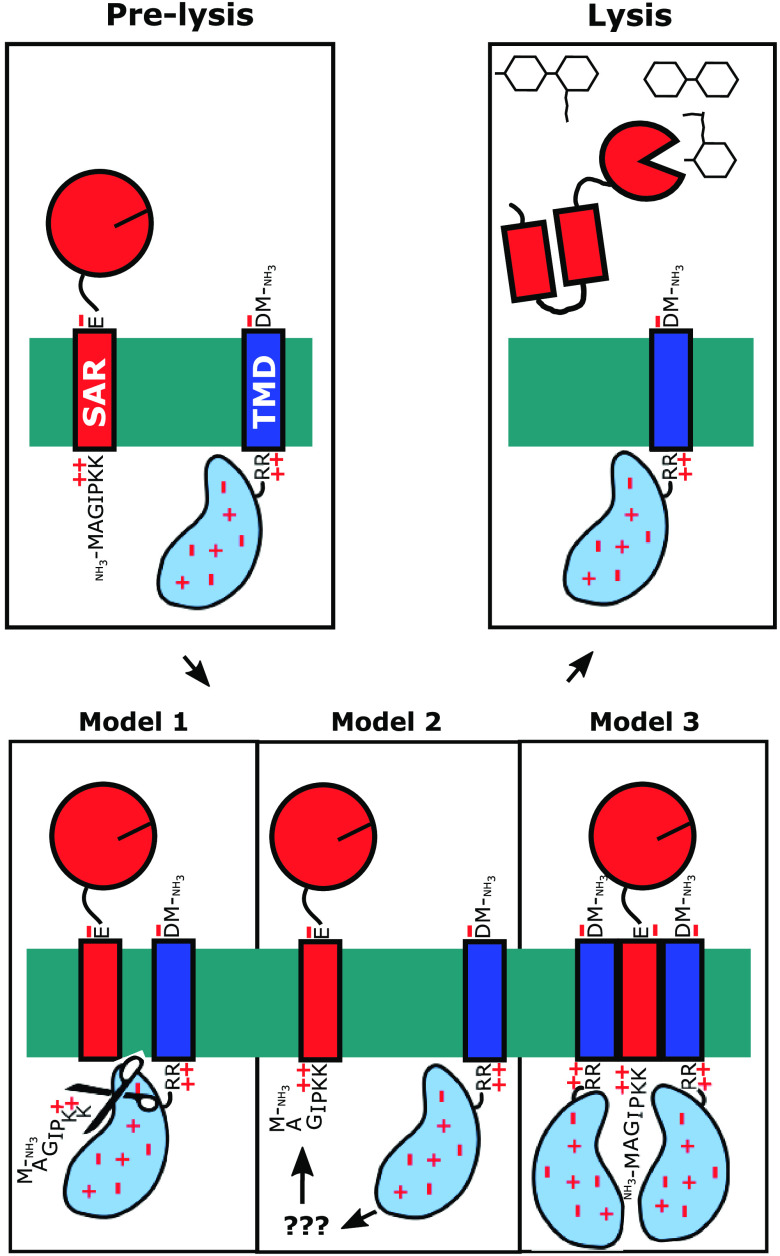
The gp22-gp25 membrane release model. (A) The SAR domain of gp22 is in the membrane and has three positively charged lysine residues. When lysis begins, gp22 and gp25 may interact via one of the three proposed models. The cytoplasmic DUF2730 gp25 domain releases the gp22 SAR domain from the membrane: (i) through proteolytic cleavage, (ii) an indirect, unknown partner, or (iii) hydrophilic channel formation. After gp22 is released from the IM, it folds into its active state, leading to degradation of the peptidoglycan and lysis.

From these considerations, several scenarios can be envisioned for releasin function (Fig. 7). One general category would be covalent modification of the cytoplasmic N terminus of gp22 to reduce the net positive charge of the first 7 residues. The simplest idea would have the cytoplasmic domain of the releasin act as a protease that would remove some or all of the cytoplasmic N-terminal segment of gp22; certainly, proteolytic enzymes which target basic residues are well known (24). Moreover, the cytoplasmic domain of gp25 has an abundance of acidic (12 of 72) and serine (9 of 72) residues, as well as 2 histidine residues. This is consistent with the catalytic and specificity-determining motifs of serine proteases that are specific for cleavage at basic residues. Other phages are known to utilize membrane-associated proteolytic cleavage at various stages in their life cycle. For example, the T4 prohead is matured at the membrane (25).

A non-covalent alternative to the protease scenario can be envisioned in which the cytoplasmic domain of gp25 would complex with the N-terminal oligopeptide of gp22 and, by virtue of closely positioned acidic residues, lower the pK_a_ of Lys6 and Lys7. Since Arg substitutions at positions 6 and 7 do not affect the gp25 requirement, the role of gp25 in this scenario would be to provide an acidic microenvironment for deprotonation of the basic N terminus of gp22. Another general model would have the releasin act as a membrane-transit chaperone by forming some sort of channel in the bilayer, allowing the charged N terminus to bypass the hydrophobic core. In this scenario, the fact that gp25 has no apparent effect on cell growth or the holin triggering makes it unlikely for a permanent pore which affects cell energetics to be formed, although this is under active investigation. Instead, gp25 would have to function in a mode operationally similar to that suggested for the TAT (twin arginine translocase) secretion apparatus, in which a channel is assembled around the secreted substrate protein (26). Interestingly, gp25 has a conserved twin arginine or arginine-lysine motif at the junction of its transmembrane and cytoplasmic domains (Fig. 6A). Although the topology of the canonical TAT signal (arginines followed by hydrophobic signal) is reversed, the gp25 twin arginine motif is, nevertheless, also at the cytoplasmic membrane interface, raising the possibility that Mu hijacks part of the TAT system for the export of gp22. Other schema can be imagined, including a catch-all idea of the participation of an unknown host factor in the release of gp22. In any case, our results suggest that gp25 can facilitate the release of highly diverse SAR endolysins, although perhaps less efficiently than for gp22 (Fig. 5). Given the topological differences between gp25 and SAR endolysins and the lack of obvious similarity in the cytoplasmic and transmembrane domains of the latter, specific interaction between the releasin and the endolysin may not play an important role in this phenomenon, which would rule out the protease model.

### DUF2730: a diverse family of Mu proteins.

gp25 is the prototype protein for a long-standing domain of unknown function, DUF2730 (Pfam entry PF10805, InterPro entry IPR020269). At the time of writing (December 2021), there are nearly 1,000 proteins harboring the DUF2730 signature in public databases, of which 38 are encoded in Mu-like (transposable) myophages and siphophages and the rest are in presumably transposable prophages in a wide range of Gram-negative hosts. The distinguishing hallmarks of transposable phages include the transposase, the protein of unknown function GemA, the Mor regulatory protein, and the portal protein (27, 28). Due to a lack of careful prophage annotation and varying levels of complete closed assemblies of host genomes containing the presumed prophage contigs, we cannot say with certainty that every instance of a DUF2730 protein is associated with a transposable phage genome. However, when we focus on the 34 genomes from active phages (usually) isolated as plaque-formers; where, in contrast to the uncharacterized prophage elements, confidence can be placed for lytic function, and we can carefully reannotate complete genomes; we see that DUF2730 proteins occur only in transposable phages. Like the Mu releasin gene, in all cases the DUF2730 gene mapped within 5 genes downstream of the endolysin cistron. Like the Mu releasin, all the phage-borne DUF2730 proteins have N-terminal TMDs with a predicted N-out, C-in topology based on the positive-inside rule (Fig. 6A) (29). However, there are three clearly distinct families as judged by alignment, only one of which (type I) has significant sequence similarity with the Mu gp25 releasin. In addition, compared in the context of the endolysin for each phage, two of the DUF2730 protein families, II and III, were associated with entirely different enzymatic classes of endolysins, transglycosylases and amidases (Fig. 6B). Moreover, other than the gp22 type, only the endolysins of type II have the weakly hydrophobic, charge-free N-terminal segments that are characteristic of N-terminal SAR domains. Thus, the operational models for the releasin proposed above would be applicable to endolysins associated with both Mu type I and II DUF2730 proteins, but not to the remaining families, which are associated with endolysins lacking SAR domains. The simplest notion would be that both the type I and II DUF2730 proteins are required for lysis, since both are associated with SAR endolysins, albeit of different enzymatic types. An important next step is to determine whether the remaining types of DUF2730 proteins are required for lysis in the context of these other Mu-like phages. Certainly, additional genetic and biochemical experiments with DUF2730 proteins are required in a wider range of systems to clarify their mechanism more broadly.

### Mu is a holin-less phage.

Heretofore, MGL systems for phages of Gram-negative hosts have been found to be tripartite, defined by the components targeting the IM, PG, and OM (Table S1 in the supplemental material). The central component of MGL systems is the holin, because holin triggering at an allele-specific time terminates morphogenesis (by collapsing the PMF) and initiates the lysis pathway involving the PG and OM components (30). Holins exhibit high genetic malleability in that single missense changes can dramatically and unpredictably advance or retard the triggering time, allowing a phage to evolve through “lysis timing space” to find the optimal length of the infection cycle (31). In addition, holin triggering can be advanced or delayed in real-time. Superinfection by most *Caudovirales* is thought to involve transient depolarization of the membrane associated with passage of the gDNA from the virion through the cytoplasmic membrane (32, 33). If a holin-mediated infection is already in progress, this would cause premature triggering, resulting in the immediate release of already matured virions and abortion of superinfection at the time of entry. In contrast, homotypic superinfection of T4-infected cells is blocked before membrane penetration and instead leads to lysis inhibition by activating the T4 antiholin (34, 35). From this perspective, the homotypic superinfection event serves as a signal of host depletion in the environment, best accommodated by postponement of lysis in favor of continued intracellular accumulation of virions (30). The work reported here shows that Mu uses a dramatically different lysis system without a traditional holin protein (Fig. 2B and C).

It is worth considering why, of all the well-studied model phages of E. coli and Salmonella, Mu might evolve a lysis system where lysis control is exerted at the SAR endolysin level. One rationale is that Mu has experienced less selection pressure for holin evolution due to its unique replication mechanism. Unlike other characterized phages, Mu copies its genome via replicative transposition during the lytic cycle (36). Typical of transposons, Mu uses transposition immunity mechanisms to avoid self-insertion (37–39). Transposition immunity in Mu is strict, within 5 kb of the genome ends, and gradually decays until becoming undetectable beyond 25 kb (40). This has significant implications for Mu’s theoretical maximum reproductive potential relative to that of non-transposing phages. Assuming a 4.6-Mb E. coli genome and perfect transposition spacing (~5 kb host DNA between each Mu genome), the maximum expected burst size for Mu should be about 460 particles. Nonetheless, the actual spacing between Mu genomes will be less uniform, as genome insertion is mostly random (41) and inhibited up to 25 kb from the ends (40). As Mu’s burst size under laboratory conditions is generally measured at ~100 to 200 particles (42), this suggests that Mu might only be capable of increasing its burst size by about 3-fold at most. In contrast, phages such as T4, λ, and N4 can delay their holin-triggering time and increase their burst sizes by as much as 10- to 100-fold (43–45). Thus, if Mu’s maximum reproductive potential is biologically constrained in this manner, it may gain little fitness benefit from the rapid temporal adaptability of holin proteins. Perhaps this has allowed Mu (and other transposable phages) to explore an unusual alternative within the evolutionary landscape of phage lysis strategies.

## MATERIALS AND METHODS

### Growth and induction of phages and bacterial strains.

Phages and bacterial strains used in this study are listed in Table 1. When plating bacterial strains, we used standard LB-agar plates supplemented with the appropriate antibiotics (ampicillin, 100 μg/mL; chloramphenicol, 10 μg/mL; kanamycin, 40 μg/mL) (46, 47). Bacteria were incubated at 30°C for Mu*c^ts^* and λ *cI857* lysogens and 37°C for non-lysogenic E. coli strains. Ara^R^ strains of MC4100 were isolated from the surviving colonies after plating cells on agar plates containing 1% (vol/vol) arabinose and re-streaking twice on 1% arabinose plates (48, 49). Strains harboring the pBAD plasmid were induced by adding arabinose to a final concentration of 0.4% (vol/vol), or repressed with 0.4% (vol/vol) glucose (50, 51). pZ-series plasmids were induced with 1 M IPTG to 1 mM. Subcultures were prepared by diluting overnight cultures inoculated from single colonies 1:250 to A_550_ ~ 0.03 in LB with antibiotics and grown at 30°C or 37°C with aeration. Bacterial growth at A_550_ was monitored using a Gilford Stasar III sipping spectrophotometer (Gilford Instrument Laboratories, Inc., Oberlin, OH), as described previously (47). Mu*c^ts^* and λ lysogens were induced by shifting the incubation temperature from 30°C to 42°C for the remainder of the experiment. For plasmid inductions in the absence of spanin function, lysis curves were recorded in the absence of divalent metal cations with vigorous agitation, unless an addition of 10 mM Mg^2+^ is stated (15). Where indicated, cultures were treated with 100% chloroform or 1 M DNP dissolved in dimethyl sulfoxide (DMSO) at the final concentrations indicated. Absorbance data were plotted with a custom “lysis-curve” package for the Python graphing library Plotly (52, 53).

**TABLE 1:** Bacteriophages and bacterial strains

Bacteriophages and bacterial strains	Genotype and features	Source
Bacteriophages		
Mu*c^ts^*	Phage Mu carrying a temp-sensitive *c^ts^62* repressor protein that is inactivated at 42°C	Laboratory stock (71)
Mu*c^ts^* 22::*cam*	Phage Mu 22::*cam*	This study
Mu*c^ts^*25::*cam*	Phage Mu 25::*cam*	This study
λ	Phage λ *cI857 bor*::*kan*	
hy21	λ *cI857 hy21*(*SRRzRz1*)_21_ *bor*::*kan*	
λ Δ(*SRRzRz1*)	λ *cI857 stf + Δ*(*SRRzRz1*) *bor*::*spec*	
		
Bacterial strains		
MG1655	E. coli K-12 *F*^−^ λ^−^ *ilvG rfb50 rph1 lacIq* ⊗*lacY tonA-* Ara^R^	Laboratory stock
MC4100	E. coli K-12 *F*^−^ *araD139* Δ(*argF-lac*)*U169 fhuA rpsL150 relA1 flbB5301 deoC1 ptsF*25 *rbsR*	Laboratory stock
MC4100 Ara^R^	E. coli K-12 *F*^−^ *araD139* Δ*argF-lac U169 fhuA rpsL150 relA1 flbB5301 deoC1 ptsF*25 *rbsR* Ara^R^	This study
MC4100 Mu*c^ts^*	MC4100 lysogenized with Mu*c^ts^62*	This study
MC4100 Mu*c^ts^* 22::*cam*	MC4100 lysogenized with Mu*c^ts^*22::*cam*	This study
MC4100 Mu*c^ts^*25::*cam*	MC4100 lysogenized with Mu*c^ts^*25::*cam*	This study
MC4100 Mu*c^ts^* Ara^R^	Ara^R^ derivative of MC4100 Mu*c^ts^*	This study
MC4100 Mu*c^ts^* 22::*cam* Ara^R^	Ara^R^ derivative of MC4100 Mu*c^ts^* 22::*cam*	This study
MC4100 Mu*c^ts^*25::*cam* Ara^R^	Ara^R^ derivative of MC4100 Mu*c^ts^* 25::*cam*	This study
MDS12	MG1655 with 12 deletions totaling 376,180 nt, including cryptic prophages, *fhuA*::*Tn10 lacI^q1^*	Laboratory stock (72)
MDS12 Mu*c^ts^*	MDS12 lysogenized with Mu*c^ts^ 62*	This study
MDS12 Mu*c^ts^* 22::*cam*	MDS12 lysogenized with Mu*c^ts^* 22::*cam*	This study
MDS12 Mu*c^ts^*25::*cam*	MDS12 lysogenized with Mu*c^ts^* 25::*cam*	This study
MDS12 λ	MDS12 lysogenized with λ *cI857 bor*::*kan*	This study
MDS12 hy21	MDS12 lysogenized with λ *cI857 hy21*(*SRRzRz1*)_21_ *bor*::*kan*	This study
MDS12 λ Δ(*SRRzRz1*)	MDS12 lysogenized with λ *cI857 stf + Δ*(*SRRzRz1*) *bor*::*spec*	This study

### Cloning and DNA constructs.

Plasmids used and constructed in this study are listed in Table 2, and primers are given in Table 3. Mu lysogens and lysates were used as the templates for cloning Mu genes. Mu genes *19*, *20*, *19 *+* 20*, *22*, and *25* were cloned in pBAD24 between its unique EcoRI and HindIII sites, or in pBAD33 between its unique KpnI and HindIII sites, with their native ribosome binding Shine-Dalgarno (S-D) sequence or with a stronger S-D sequence (AGGAGG) (50, 54). Alleles encoding gp22 or gp25 were constructed with the c-*myc* or His6 oligopeptide tags at the 3′ end of the gene, respectively. Gene *22* and *25* mutants were constructed by using a Quick-Change site-directed mutagenesis kit (Agilent, Santa Clara, CA) on pBAD33-gp22-c-*myc* or pBAD24-gp25-his, respectively.

**TABLE 2:** Plasmids used in this study

Plasmid	Description	Source
pKD46	(λ) Red recombinase expression system	Laboratory stock (73)
pBAD33	pACYC184-derived plasmid with P_BAD_ promoter; *araC cat*	Laboratory stock (50)
pBAD24	pBR322-derived plasmid with P_BAD_ promoter; *araC bla*	Laboratory stock (50)
pBAD18-Kan	pBR322-derived plasmid with P_BAD_ promoter; *araC neo*	Laboratory stock (50)
pZE12	pZE12-luc	Laboratory stock (74)
pBAD33-gp22-c-myc	pBAD33 with Mu gp22-c-myc inserted between the KpnI and HindIII sites after Shine Dalgarno AGGAGG	This study
pBAD33-gp22-c-myc-K6A	K6A mutation in gp22-c-myc	This study
pBAD33-gp22-c-myc-K7A	K7A mutation in gp22-c-myc	This study
pBAD33-gp22-c-myc-K9A	K9A mutation in gp22-c-myc	This study
pBAD33-gp22-c-myc-K6R	K6R mutation in gp22-c-myc	This study
pBAD33-gp22-c-myc-K7R	K7R mutation in gp22-c-myc	This study
pBAD33-gp22-c-myc-K9R	K9R mutation in gp22-c-myc	This study
pBAD33-gp22-c-myc-K6A-K7A	K6A and K9A mutations in gp22-c-myc	This study
pBAD33-gp22-c-myc-K7A-K9A	K7A and K9A mutations in gp22-c-myc	This study
pBAD33-gp22-c-myc-K6R-K7R	Derived from pBAD33-gp22-c-myc-K6R, added K7R mutations in gp22-c-myc	This study
pBAD24-gp*19*	pBAD24 with Mu gp*19* under Shine Dalgarno AGGAGG	This study
pBAD33-gp*20*	pBAD33 with Mu gp*20* under Shine Dalgarno AGGAGG	This study
pBAD24-gp*19*+gp*20*	pBAD24 with Mu gp*19* and gp*20* inserted with native RBS	This study
pBAD24-gp25-his	pBAD24 plasmid with Mu gp25-his cloned into EcoRI and HindIII sites under Shine Dalgarno AGGAGG	This study
pBAD33-gp25-his	pBAD33 plasmid with Mu gp25-his under Shine Dalgarno AGGAGG	This study
pBAD18-Kan-Mu-Pm-gp25	pBAD18-Kan with the Mu middle promoter P_m_ through gp25	This study
pBAD18-Kan-Mu-gp*18*-gp25	Derived from pBAD18-Kan-Mu Pm-gp25, P_m_ deleted	This study
pBAD18-Kan-Mu-gp*18*-gp25-ΔP_lys_	Derived from pBAD18-Kan-Mu gp*18*-gp25, P_lys_ deleted, placing all genes under P_BAD_ control	This study
pBAD18-Kan-Mu-gp*18*-gp25-gp22-*E37A*	Derived from pBAD18-Kan-Mu-gp*18*-gp25-ΔP_lys_, inactivating catalytic domain mutation E37A in gp22	This study
pBAD18-Kan-Mu-gp22-gp25	Derived from pBAD18-Kan-Mu-gp*18*-gp25-ΔP_lys_, genes 18–21 deleted	This study
pBAD18-Kan-Mu-gp22-gp25-gp*23.1_am_*	Derived from pBAD18-Kan-Mu-gp22-gp25, W65 amber mutation in gp*23.1*	This study
pBAD33-λ-*RzRz1*	pBAD33 with λ spanins *RzRz1* subcloned into the KpnI and HindIII sites	Laboratory stock
pBAD24-21*-S68*	pBAD24 with phage 21 *S68* pinholin	This study
pBAD24-21*-S68_am_*	pBAD24-21-*S68* with amber allele at V43	This study
pBAD24-λ-*S105*	pBAD24 with λ *S105* holin	This study
pZE12-gp22	pZE12 with Mu gp22 insert	This study
pZE12-21-*R*-c-myc	pZE12 with phage 21 SAR endolysin insert with a C-terminal *myc* tag (EQKLISEEDL)	Laboratory stock (11)
pZE12-*933W-R*	pZE12 with phage 933W SAR endolysin insert	Laboratory stock (11)
pZE12-*Bcep*22*-R*	pZE12 with phage Bcep22 SAR endolysin insert	Laboratory stock (11)

**TABLE 3:** Oligonucleotides used in this study

Primer name	5′–3′ Sequence	Purpose
MuSAR For	CATCAGAGAATTTTTTCAGGGAAGC	Cam knockout of 22
MuSAR Cam Rev	CTTTTACTGGCGAAGGTCATCCGCCCCCCTGATATTCCAGATTGCCATTTCATGGGAATTAGCCATGGTCC	Cam knockout of 22
Mu gp25 Cam For	GGTGCCGTGGAGCAGAATAATGGATTTGATTTCAGTTTTAGCGTTATGGGTGTAGGCTGGAGCTGCTTC	Cam knockout of 25
Mu gp25 Cam Rev	CGACGGTCTTCGGTCAGAATATCGTTAATCATGAATTACTCCGGTTTACTGATGGGAATTAGCCATGGTCC	Cam knockout of 25
Mu gp25 EcoRI For	GGAATTCATGGATTTGATTTCAGTTTTAGC	Clone gp25 with ssd
gp25 C-his Rev HindIII	CCCAAGCTTTCAATGATGATGGTGGTGGTGACCTCCTG	Clone gp25 with C-terminal his tag
Mu gp22 pBAD33 For KpnI	GGGGTACCTTCAGGGAAGCATGATGG	Clone gp22 with ssd
gp22 KpnI Strong SD For	GGGGTACCAGGAGGAATTCATGG	Clone gp22 with osd
Mu gp22 pBAD33Rev HindIII	CCCAAGCTTTTACTGGCGAAGGTCATC	Clone untagged gp22
pBAD24 gp22 C-myc QC For	CAGAAGAGGATCTGTAAAAGCTTGGCTGTTTTGG	Clone gp22 with C-terminal c-*myc* tag
pBAD24 gp22 C-myc QC Rev	AGATGAGTTTTTGTTCCTGGCGAAGGTCATCCG	Clone gp22 with C-terminal c-*myc* tag
K6A gp22 Forward	GGATACCAGCAAAACTGAAAGCCGCACTGC	gp22 mutagenesis
K6A gp22 Rev Comp	TGCGGCTTTCAGTTTTGCTGGTATCCCCG	gp22 mutagenesis
K7A gp22 Forward	GGATACCAAAAGCACTGAAAGCCGCACTGC	gp22 mutagenesis
K7A gp22 Rev Comp	GGCTTTCAGTGCTTTTGGTATCCCCGCC	gp22 mutagenesis
K9A gp22 Forward	GGATACCAAAAAAACTGGCAGCCGCACTG	gp22 mutagenesis
K9A gp22 Rev Comp	CAGTGCGGCTGCCAGTTTTTTTGGTATCCCC	gp22 mutagenesis
K6R gp22 Forward	GGATACCAAGAAAACTGAAAGCCGCAC	gp22 mutagenesis
K6R gp22 Rev Comp	CTTTCAGTTTTCTTGGTATCCCCGCC	gp22 mutagenesis
K7R gp22 Forward	GGATACCAAAAAGACTGAAAGCCGC	gp22 mutagenesis
K7R gp22 Rev Comp	GCTTTCAGTCTTTTTGGTATCCCCGC	gp22 mutagenesis
K9R gp22 Forward	AAAAAAACTGAGAGCCGCACTGCTGG	gp22 mutagenesis
K9R gp22 Rev Comp	CAGTGCGGCTCTCAGTTTTTTTGGTATCCC	gp22 mutagenesis
K6A K7A gp22 Forward	CGGGGATACCAGCAGCCCTGAAAGCCGCACTGC	gp22 mutagenesis
K6A K7A gp22 Rev Comp	CGGCTTTCAGGGCTGCTGGTATCCCCGCCATG	gp22 mutagenesis
K7A K9A gp22 Forward	CATGGCGGGGATACCAAAAGCACTGGCAGCCGCACTG	gp22 mutagenesis
K7A K9A gp22 Rev Comp	CAGTGCGGCTGCCAGTGCTTTTGGTATCCCCGCC	gp22 mutagenesis
Mu gp22 K6R K7R F	CGTAGACTGAAAGCCGCAC	gp22 mutagenesis
Mu gp22 ssd P5 R	TGGTATCCCCGCCATGAATTC	gp22 mutagenesis
Mu gp19 For EcoRI	GGAATTCATGTCTGAGCGTTCTGC	Clone gp*19*
Mu gp19 Rev HindIII	CCCAAGCTTTTACCCGAACAGACG	Clone gp*19*
Mu gp*20* ssd KpnI F	acgtggtaccaggaggaattcatgtacagaaaattcagtg	Clone gp*20*
Mu gp*20* HindIII R	agccaagcttttaacagacagaaagatacaccacggc	Clone gp*20*
Mu gp*20*-pBAD F	GATAACCATCAGTGCCGTGGTGTATCTTTCTGTCTGTTAAAAGCTTGGCTGTTTTGGC	Clone gp*19*+gp*20*
Mu gp*19*-pBAD R	GGCCACTGACGAGCAGAACGCTCAGACATAATGTTTCTCCTCTAGAGGATCCCCGGGTAC	Clone gp*19*+gp*20*
Mu gp*19* F	GGAGAAACATTATGTCTGAG	Clone gp*19*+gp*20*
Mu gp*20* R	TTAACAGACAGAAAGATACAC	Clone gp*19*+gp*20*
Mu Pm nt9163 F	ttctgtaaacagtaaagccggttaatccggc	Clone Mu18–25
Mu gp25 end R	tcatgaattactccggtttactgcg	Clone Mu18–25
Mu ΔP_m_-pBAD18 F	CGGGGAAAATCATCATGGG	Delete Mu P_m_
Mu ΔP_m_-pBAD18 R	CCGAGCTCGAATTCGCTAG	Delete Mu P_m_
Mu gp21 end R	TTATTTTGGGGTTTCAGGTGAGAACAGA	Delete Mu P_lys_ in gp18-gp25 ΔP_m_ clone
Mu gp22 pBAD F	AGGGAAGCATGATGGCGGGGATAC	Delete Mu P_lys_ in gp18-gp25 ΔP_m_ clone
Mu gp22 pBAD F	AGGGAAGCATGATGGCGGGGATAC	Delete Mu gp18-gp21 in gp18-gp25 ΔP_m_ ΔP_lys_ clone
pBAD18-Kan R	CCGAGCTCGAATTCGCTAG	Delete Mu gp18-gp21 in gp18-gp25 ΔP_m_ ΔP_lys_ clone
Mu Mup23.1 W65am F	CCCCTGACGTAGGGAGCATCC	Clone gp22-gp25 23.1-am
Mu Mup23.1 Q61 R	CTGTGGCATGGGCGGAAC	Clone gp22-gp25 23.1-am
Mu gp22 E37A F	ATCCACCTGGCAAATATCGCTTATATG	gp22 catalytic domain knockout E37A
Mu gp22 L33 R	CAGAGACTGACGGGTCAT	gp22 catalytic domain knockout E37A
21 S68 pBAD24 F	TGGGCTAGCAGGAGGAATTCATGGACAAAATCTCAACTGGCATTG	Clone S68 and S68am
21 S68 pBAD24 R	CCGCCAAAACAGCCAAGCTTTTATTCACCTCTCGCAGCCTT	Clone S68 and S68am
pBAD24 21 S68 F	AGGCTGCGAGAGGTGAATAAAAGCTTGGCTGTTTTGGCG	Clone S68 and S68am
pBAD24 21 S68 R	CCAGTTGAGATTTTGTCCATGAATTCCTCCTGCTAGCCCA	Clone S68 and S68am
λ pBAD24 F	TGGGCTAGCAGGAGGAATTCATGCCAGAAAAACATGACCTGTT	Clone S105
λ pBAD24 R	CCGCCAAAACAGCCAAGCTTTTATTGATTTCTACCATCTTCTACTCCGGC	Clone S105
pBAD24 λ S105 F	AAGATGGTAGAAATCAATAAAAGCTTGGCTGTTTTGGCG	Clone S105
pBAD24 λ S105 R	AGGTCATGTTTTTCTGGCATGAATTCCTCCTGCTAGCCCA	Clone S105
pZE12 XbaI F	TCTAGAGGCATCAAATAAAACG	Clone untagged gp22
pZE12 KpnI R	GGTACCTTTCTCCTCTTTAATG	Clone untagged gp22
Mu gp22-pZE12 F	ACCGAATTCATTAAAGAGGAGAAAGGTACCATGGCGGGGATACCAAAAAAACTGAAAGCC	Clone untagged gp22
Mu gp22-pZE12 R	GAGCCTTTCGTTTTATTTGATGCCTCTAGATTACTGGCGAAGGTCATCCGC	Clone untagged gp22

Construction of the λ lysogen lacking a lysis cassette relied on recombination between λ *stf cI857* and a pLambchops plasmid. The pLambchops plasmid was missing the entire λ lysis cassette but retained upstream and downstream homology to the phage late region. pLambchops was generated by inverse PCR on a spectinomycin-resistant pRE RzRz1 vector generated from the combination of a commercially synthesized gene block with the spanin-containing vector.

### Construction of Mu knockout mutants.

Construction of Mu*c^ts^* deletion-substitutions was performed as described previously (48, 55). Briefly, flanking regions of the target genes (*19*, *20*, *22*, and *25*) were attached to the *cat* gene via PCR using primers listed in Table 3. Each construct retained the bases encoding ~10 residues at the ends so as not to disrupt overlapping upstream and downstream features. Next, 10 to 100 ng of purified linear dsDNA PCR products was digested with DpnI and transferred into MC4100 Ara^R^ cells carrying Red helper plasmid pKD46 by electroporation. Prior to electroporation, MC4100 Ara^R^ cells were grown at 30°C in LB supplied with Amp and l-arabinose (Ara) and then resuspended in 10% glycerol. Cam-resistant colonies were selected. The *cat* insertion was confirmed by PCR and Sanger sequencing with Eton Biosciences (San Diego, CA).

Mu phage stocks collected from induced prophages were sterilized and used to infect MDS12 cultures at a low multiplicity of infection, then selected for Cam resistance via plating at 30°C or tested for temperature sensitivity for the Mu*c^ts^* strain. The lysogens were subjected to next-generation sequencing to determine the location and number of insertions.

### Microscopy and visualization of cells.

To visualize each sample, 1 μL of culture was placed on a glass slide under a coverslip. Snapshots were acquired with an Axiocam 702 mono camera mounted on a Zeiss Axio Observer 7 inverted microscope equipped with plan-neofluar 4×/0.75 NA Ph2 and alpha plan-apochromat 100×/1.46 oil (UV) Ph3 oil M27 objectives. A glass slide was not used when recording videos of motile cells: cell culture was applied directly to a coverslip and left exposed to allow for air exchange and free cell movement. Images and time-series videos were processed by minor brightness/contrast adjustments within the Carl Zeiss Zen 2.3 imaging software.

### Recovery of 2,4-dinitrophenol-treated cells.

MG1655 cells containing pBAD plasmids were treated with DNP at a final concentration of 2 mM 60 min after plasmid induction. Ten min later, a 5-mL aliquot of cells was transferred to a 50-mL conical tube and centrifuged at 5,000 × *g* for 10 min. After the supernatant was discarded, cells were resuspended in fresh LB with the appropriate antibiotics and 0.4% arabinose and transferred to a 250-mL flask. Recovery incubation proceeded at 37°C in a water bath with aeration.

### Protein structural models.

Protein covariance was assessed locally with the EVcouplings Python pipelines using the UniRef100 database (February 2020 release) for alignment and PSIPRED BLAST (56, 57). Evolutionary couplings for gp22 (both full-length and range restricted to SAR residues 5 to 30) and gp25 (full-length or cytoplasmic residues 25 to 99) were calculated in the monomer pipeline, using default parameters. Protein models were visualized using UCSF ChimeraX (58). Transmembrane topology diagrams were generated using TOPO2 (Johns S.J., TOPO2, transmembrane protein display software available from http://www.sacs.ucsf.edu/TOPO2/).

### SDS-PAGE and Western blotting.

Whole protein samples were prepared from culture samples taken at specific time points after induction by addition to ice-cold 10% (vol/vol) trichloroacetic acid. Pelleted protein samples were then washed with cold acetone before resuspension in nonreducing Laemmli buffer. Electrophoresis on Novex 16% Tricine gels (Thermo Fisher Scientific, Waltham, MA) was performed according to manufacturer recommendations. Proteins were transferred to a 0.2-μm polyvinylidene difluoride membrane by the iBlot2 system with a pre-programmed protocol (20 V for 1 min, 23 V for 4 min, 25 V for 1 to 2 min). After blocking with 4% (wt/vol) milk–Tris-buffered saline; His-tagged and c-*myc* tagged proteins were detected separately. Antibodies and substrate were acquired from Thermo Fisher Scientific (Waltham, MA). The mouse α-His primary antibody was used at a 1:2,000 dilution, and the secondary goat-anti-mouse Alexa Fluor 680 was used at a 1:20,000 dilution. α-c-*myc* (9E10) mouse monoclonal was used at a dilution of 1:1,000 overnight, followed by secondary antibody (goat-anti-mouse horseradish peroxidase) at 1:1,000, with SuperSignal West Femto Maximum Sensitivity Chemiluminescent Substrate (Thermo Fisher) for detection. Blots were scanned on Amersham Imager 600 RGB (GE Healthcare, Chicago, LA) and Bio-Rad-ChemiDoc machines, respectively, and analyzed using LI-COR ImageStudioLite version 4.0.21 software.

### Cell shape counting assay.

MG1655 cells were subcultured 1:250 from an overnight into 250-mL flasks containing 25 mL LB, appropriate antibiotics, and 10 mM Mg^2+^. Flasks were incubated at 37°C with aeration in a water bath. At A_550_ ~ 0.2, the pBAD vector was induced with addition of arabinose to 0.4% (T = 0). Thirty minutes after pBAD vector induction (T = 30), aliquots were taken and placed on ice for microscopy visualization. Forty minutes after pBAD vector induction (T = 40), 1 M IPTG was added to a final concentration of 1 mM to induce the pZE12 vector. At T = 50, aliquots were taken and placed on ice for microscopy visualization. Counting of “round”- versus “rod”-shaped cells was accomplished with assistance from a custom analysis program within the proprietary Zen 2.3 software.

### Protein bioinformatic analyses.

The Mu gp25 amino acid sequence was searched in the InterPro 86.0 June 2021 database release and analyzed by BLASTp against bacteria and phage sequences in the NCBI nonredundant database (59, 60). Thirty-eight *Caudovirales* phages are listed with a protein containing the DUF2730 domain (equivalent to Pfam entry PF10805, InterPro entry IPR020269). Of these, 3 metagenome entries and 1 incomplete genome were excluded, for 34 complete records sequenced from active phages retrieved from the NCBI genome record and inspected in Artemis or the Center for Phage Technology Galaxy and Apollo web instance (https://cpt.tamu.edu/galaxy-pub) (61, 62). Full information on these phages, including accession numbers, taxonomic classification, and their hosts, is given in Table S1. In 33 cases, the phage endolysins was identified within 10 genes up- or downstream by analyzing InterProScan for domains known to be associated with endolysins. The endolysin for *Vibrio* phage Martha 12B12 was not identifiable by this bioinformatic method; instead, that genome was aligned with *Vibrio* phage 1.028.O._10N.286.45.B6 using EasyFig (63), then by using HHPred through their web interface (20). Manual inspection of synteny and analyses were conducted with TMHMM, Phobius, and InterProScan in the Center for Phage Technology Galaxy instance to predicts lysozyme and transmembrane domains for phage Martha (64–67). Full protein sets were aligned in CLC Genomics Workbench (Qiagen, Redwood City, CA). In addition to the tools mentioned above, predicted secondary structures for all lysis proteins were inspected using HHPred in the MPI Bioinformatics Toolkit (20).

### Figure preparation.

Graphics for all figures were combined for display in Inkscape 1.0 (68).

## References

[1] Cahill J, Young R. 2019. Phage lysis: multiple genes for multiple barriers. Adv Virus Res 103:33–70. doi:10.1016/bs.aivir.2018.09.003. 30635077 PMC6733033

[2] Kongari R, Rajaure M, Cahill J, Rasche E, Mijalis E, Berry J, Young R. 2018. Phage spanins: diversity, topological dynamics and gene convergence. BMC Bioinformatics 19:326. doi:10.1186/s12859-018-2342-8. 30219026 PMC6139136

[3] Holt A, Cahill J, Ramsey J, Martin C, O’Leary C, Moreland R, Maddox LT, Galbadage T, Sharan R, Sule P, Cirillo JD, Young R. 2022. Phage-encoded cationic antimicrobial peptide required for lysis. J Bacteriol 204:e00214-21. doi:10.1128/JB.00214-21. PMC8765421 34339297

[4] Symonds N, Toussaint A, Howe M. 1987. Phage Mu. Cold Spring Harbor Laboratory, Cold Spring Harbor, NY.

[5] Faelen M, Toussaint A. 1973. Isolation of conditional defective mutants of temperate phage Mu-1 and deletion mapping of the Mu-1 prophage. Virology 54:117–124. doi:10.1016/0042-6822(73)90121-9. 4576742

[6] Howe MM. 1973. Prophage deletion mapping of bacteriophage Mu-1. Virology 54:93–101. doi:10.1016/0042-6822(73)90118-9. 4351616

[7] Howe MM, O'Day KJ, Schultz DW. 1979. Isolation of mutations defining five new cistrons essential for development of bacteriophage Mu. Virology 93:303–319. doi:10.1016/0042-6822(79)90235-6. 452408

[8] Morgan GJ, Hatfull GF, Casjens S, Hendrix RW. 2002. Bacteriophage Mu genome sequence: analysis and comparison with Mu-like prophages in *Haemophilus*, *Neisseria* and *Deinococcus*. J Mol Biol 317:337–359. doi:10.1006/jmbi.2002.5437. 11922669

[9] Vermassen A, Leroy S, Talon R, Provot C, Popowska M, Desvaux M. 2019. Cell wall hydrolases in bacteria: insight on the diversity of cell wall amidases, glycosidases and peptidases toward peptidoglycan. Front Microbiol 10:331. doi:10.3389/fmicb.2019.00331. 30873139 PMC6403190

[10] Samanta S, Sharma A, Saha A, Singh M, Das A, Bhattacharya M, Saha R, Lee S-S, Chakraborty C. 2021. The bacteriophage Mu lysis system: a new mechanism of host lysis? Biocell 45:1175–1186. doi:10.32604/biocell.2021.015537.

[11] Kuty GF. 2011. SAR endolysin regulation in dsDNA phage lysis of Gram-negative hosts. Doctoral Dissertation. Texas A&M University, College Station, TX.

[12] Xu M, Struck DK, Deaton J, Wang I-N, Young R. 2004. A signal-arrest-release sequence mediates export and control of the phage P1 endolysin. Proc Natl Acad Sci USA 101:6415–6420. doi:10.1073/pnas.0400957101. 15090650 PMC404059

[13] Park T, Struck DK, Dankenbring CA, Young R. 2007. The pinholin of lambdoid phage 21: control of lysis by membrane depolarization. J Bacteriol 189:9135–9139. doi:10.1128/JB.00847-07. 17827300 PMC2168629

[14] Young R. 2014. Phage lysis: three steps, three choices, one outcome. J Microbiol 52:243–258. doi:10.1007/s12275-014-4087-z. 24585055 PMC4012431

[15] Berry J, Rajaure M, Pang T, Young R. 2012. The spanin complex is essential for lambda lysis. J Bacteriol 194:5667–5674. doi:10.1128/JB.01245-12. 22904283 PMC3458670

[16] Young R. 2013. Phage lysis: do we have the hole story yet? Curr Opin Microbiol 16:790–797. doi:10.1016/j.mib.2013.08.008. 24113139 PMC3848059

[17] Mathee K, Howe MM. 1993. The bacteriophage Mu middle operon: essential and nonessential functions. Virology 196:712–721. doi:10.1006/viro.1993.1528. 8372443

[18] Pang T, Fleming TC, Pogliano K, Young R. 2013. Visualization of pinholin lesions *in vivo*. Proc National Acad Sci USA 110:E2054-63. doi:10.1073/pnas.1222283110. PMC3670314 23671069

[19] Babu K, Arulandu A, Sankaran K. 2018. The structure of DLP12 endolysin exhibiting alternate loop conformation and comparative analysis with other endolysins. Proteins 86:210–217. doi:10.1002/prot.25428. 29179254

[20] Zimmermann L, Stephens A, Nam S-Z, Rau D, Kübler J, Lozajic M, Gabler F, Söding J, Lupas AN, Alva V. 2018. A completely reimplemented MPI bioinformatics toolkit with a new HHpred server at its core. J Mol Biol 430:2237–2243. doi:10.1016/j.jmb.2017.12.007. 29258817

[21] Sun Q, Kuty GF, Arockiasamy A, Xu M, Young R, Sacchettini JC. 2009. Regulation of a muralytic enzyme by dynamic membrane topology. Nat Struct Mol Biol 16:1192–1194. doi:10.1038/nsmb.1681. 19881499 PMC3075974

[22] Oliveira H, Melo LDR, Santos SB, Nóbrega FL, Ferreira EC, Cerca N, Azeredo J, Kluskens LD. 2013. Molecular aspects and comparative genomics of bacteriophage endolysins. J Virol 87:4558–4570. doi:10.1128/JVI.03277-12. 23408602 PMC3624390

[23] Gontijo MTP, Vidigal PMP, Lopez MES, Brocchi M. 2021. Bacteriophages that infect Gram-negative bacteria as source of signal-arrest-release motif lysins. Res Microbiol 172:103794. doi:10.1016/j.resmic.2020.103794. 33347948

[24] Vandermarliere E, Mueller M, Martens L. 2013. Getting intimate with trypsin, the leading protease in proteomics: trypsin in proteomics. Mass Spectrom Rev 32:453–465. doi:10.1002/mas.21376. 23775586

[25] Black LW, Showe MK. 1983. Bacteriophage T4, p 219–245. *In* Matthews CK, Kutter EM, Mosig G, Berget PB (ed), Morphogenesis of the T4 head. American Society for Microbiology, Washington, DC.

[26] Frain KM, Robinson C, van Dijl JM. 2019. Transport of folded proteins by the Tat system. Protein J 38:377–388. doi:10.1007/s10930-019-09859-y. 31401776 PMC6708511

[27] Hulo C, Masson P, Mercier PL, Toussaint A. 2015. A structured annotation frame for the transposable phages: a new proposed family “*Saltoviridae*” within the *Caudovirales*. Virology 477:155–163. doi:10.1016/j.virol.2014.10.009. 25500185

[28] Toussaint A, Gijsegem FV. 2018. Extension of the transposable bacterial virus family: two genomic organisations among phages and prophages with a Tn552-related transposase. Res Microbiol 169:495–499. doi:10.1016/j.resmic.2017.11.002. 29158161

[29] von Heijne G. 1992. Membrane protein structure prediction. Hydrophobicity analysis and the positive-inside rule. J Mol Biol 225:487–494. doi:10.1016/0022-2836(92)90934-c. 1593632

[30] Young R. 2002. Bacteriophage holins: deadly diversity. J Mol Microb Biotechnol 4:21–36. 11763969

[31] Zheng Y, Struck DK, Dankenbring CA, Young R. 2008. Evolutionary dominance of holin lysis systems derives from superior genetic malleability. Microbiology (Reading) 154:1710–1718. doi:10.1099/mic.0.2008/016956-0. 18524925 PMC5995320

[32] Labedan B, Letellier L. 1981. Membrane potential changes during the first steps of coliphage infection. Proc Natl Acad Sci USA 78:215–219. doi:10.1073/pnas.78.1.215. 7017710 PMC319022

[33] Letellier L, Boulanger P. 1989. Involvement of ion channels in the transport of phage DNA through the cytoplasmic membrane of *E. coli*. Biochimie 71:167–174. doi:10.1016/0300-9084(89)90147-8. 2470417

[34] Ramanculov E, Young R. 2001. An ancient player unmasked: T4 rI encodes a t-specific antiholin. Mol Microbiol 41:575–583. doi:10.1046/j.1365-2958.2001.02491.x. 11532126

[35] Hays SG, Seed KD. 2020. Dominant *Vibrio cholerae* phage exhibits lysis inhibition sensitive to disruption by a defensive phage satellite. Elife 9:e53200. doi:10.7554/eLife.53200. 32329714 PMC7182436

[36] Harshey RM. 1984. Transposition without duplication of infecting bacteriophage Mu DNA. Nature 311:580–581. doi:10.1038/311580a0. 6090947

[37] Darzins A, Kent NE, Buckwalter MS, Casadaban MJ. 1988. Bacteriophage Mu sites required for transposition immunity. Proc Natl Acad Sci USA 85:6826–6830. doi:10.1073/pnas.85.18.6826. 2842794 PMC282071

[38] Adzuma K, Mizuuchi K. 1988. Target immunity of Mu transposition reflects a differential distribution of Mu B protein. Cell 53:257–266. doi:10.1016/0092-8674(88)90387-X. 2965985

[39] Ge J, Lou Z, Harshey RM. 2010. Immunity of replicating Mu to self-integration: a novel mechanism employing MuB protein. Mob Dna 1:8. doi:10.1186/1759-8753-1-8. 20226074 PMC2837660

[40] Manna D, Higgins NP. 1999. Phage Mu transposition immunity reflects supercoil domain structure of the chromosome. Mol Microbiol 32:595–606. doi:10.1046/j.1365-2958.1999.01377.x. 10320581

[41] Walker DM, Harshey RM. 2020. Deep sequencing reveals new roles for MuB in transposition immunity and target-capture, and redefines the insular Ter region of *E. coli*. Mob Dna 11:26. doi:10.1186/s13100-020-00217-9. 32670425 PMC7350765

[42] Waggoner BT, González NS, Taylor AL. 1974. Isolation of heterogeneous circular DNA from induced lysogens of bacteriophage Mu-1. Proc Natl Acad Sci USA 71:1255–1259. doi:10.1073/pnas.71.4.1255. 4598297 PMC388204

[43] Reader RW, Siminovitch L. 1971. Lysis defective mutants of bacteriophage lambda: genetics and physiology of S cistron mutants. Virology 43:607–622. doi:10.1016/0042-6822(71)90286-8. 4940968

[44] Doermann AH. 1948. Lysis and lysis inhibition with *Escherichia coli* bacteriophage. J Bacteriol 55:257–276. doi:10.1128/jb.55.2.257-276.1948. 16561455 PMC518436

[45] Schito GC, Molina AM, Pesce A. 1967. Lysis and lysis inhibition with N4 coliphage. Giornale di Microbiologia 15:229–244.

[46] Moussa SH, Lawler JL, Young R. 2014. Genetic dissection of T4 lysis. J Bacteriol 196:2201–2209. doi:10.1128/JB.01548-14. 24706740 PMC4054191

[47] To KH, Dewey J, Weaver J, Park T, Young R. 2013. Functional analysis of a class I holin, P2 Y. J Bacteriol 195:1346–1355. doi:10.1128/JB.01986-12. 23335412 PMC3592007

[48] Bolhuis A, Bogsch EG, Robinson C. 2000. Subunit interactions in the twin-arginine translocase complex of *Escherichia coli*. FEBS Lett 472:88–92. doi:10.1016/s0014-5793(00)01428-9. 10781811

[49] Englesberg E, Anderson R, Weinberg R, Lee N, Hoffee P, Huttenhauer G, Boyer H. 1962. L-Arabinose-sensitive, l-ribulose 5-phosphate 4-epimerase-deficient mutants of *Escherichia coli*. J Bacteriol 84:137–146. doi:10.1128/jb.84.1.137-146.1962. 13890280 PMC277779

[50] Guzman LM, Belin D, Carson MJ, Beckwith J. 1995. Tight regulation, modulation, and high-level expression by vectors containing the arabinose PBAD promoter. J Bacteriol 177:4121–4130. doi:10.1128/jb.177.14.4121-4130.1995. 7608087 PMC177145

[51] Wang I-N, Deaton J, Young R. 2003. Sizing the holin lesion with an endolysin-beta-galactosidase fusion. J Bacteriol 185:779–787. doi:10.1128/JB.185.3.779-787.2003. 12533453 PMC142811

[52] Inc. PT. 2015. Collaborative data science publisher: Plotly Technologies Inc., Montréal, Quebec, Canada. Available from https://plot.ly.

[53] Chamblee J. 2021. jakechamblee/lysis-curve: 1.12 (1.12.). Zenodo. Available from 10.5281/zenodo.5514286.

[54] Bläsi U, Young R. 1996. Two beginnings for a single purpose: the dual-start holins in the regulation of phage lysis. Mol Microbiol 21:675–682. doi:10.1046/j.1365-2958.1996.331395.x. 8878031

[55] Grundling A, Smith DL, Bläsi U, Young R. 2000. Dimerization between the holin and holin inhibitor of phage lambda. J Bacteriol 182:6075–6081. doi:10.1128/JB.182.21.6075-6081.2000. 11029427 PMC94741

[56] Hopf TA, Green AG, Schubert B, Mersmann S, Schärfe CPI, Ingraham JB, Toth-Petroczy A, Brock K, Riesselman AJ, Palmedo P, Kang C, Sheridan R, Draizen EJ, Dallago C, Sander C, Marks DS. 2019. The EVcouplings Python framework for coevolutionary sequence analysis. Bioinformatics 35:1582–1584. doi:10.1093/bioinformatics/bty862. 30304492 PMC6499242

[57] Hopf TA, Colwell LJ, Sheridan R, Rost B, Sander C, Marks DS. 2012. Three-dimensional structures of membrane proteins from genomic sequencing. Cell 149:1607–1621. doi:10.1016/j.cell.2012.04.012. 22579045 PMC3641781

[58] Pettersen EF, Goddard TD, Huang CC, Couch GS, Greenblatt DM, Meng EC, Ferrin TE. 2004. UCSF Chimera: a visualization system for exploratory research and analysis. J Comput Chem 25:1605–1612. doi:10.1002/jcc.20084. 15264254

[59] Camacho C, Coulouris G, Avagyan V, Ma N, Papadopoulos J, Bealer K, Madden TL. 2009. BLAST+: architecture and applications. BMC Bioinformatics 10:421. doi:10.1186/1471-2105-10-421. 20003500 PMC2803857

[60] Mitchell AL, Attwood TK, Babbitt PC, Blum M, Bork P, Bridge A, Brown SD, Chang H-Y, El-Gebali S, Fraser MI, Gough J, Haft DR, Huang H, Letunic I, Lopez R, Luciani A, Madeira F, Marchler-Bauer A, Mi H, Natale DA, Necci M, Nuka G, Orengo C, Pandurangan AP, Paysan-Lafosse T, Pesseat S, Potter SC, Qureshi MA, Rawlings ND, Redaschi N, Richardson LJ, Rivoire C, Salazar GA, Sangrador-Vegas A, Sigrist CJA, Sillitoe I, Sutton GG, Thanki N, Thomas PD, Tosatto SCE, Yong S-Y, Finn RD. 2018. InterPro in 2019: improving coverage, classification and access to protein sequence annotations. Nucleic Acids Res 47:D351–D360. doi:10.1093/nar/gky1100. PMC6323941 30398656

[61] Ramsey J, Rasche H, Maughmer C, Criscione A, Mijalis E, Liu M, Hu JC, Young R, Gill JJ. 2020. Galaxy and Apollo as a biologist-friendly interface for high-quality cooperative phage genome annotation. PLoS Comput Biol 16:e1008214. doi:10.1371/journal.pcbi.1008214. 33137082 PMC7660901

[62] Carver T, Harris SR, Berriman M, Parkhill J, McQuillan JA. 2012. Artemis: an integrated platform for visualization and analysis of high-throughput sequence-based experimental data. Bioinformatics 28:464–469. doi:10.1093/bioinformatics/btr703. 22199388 PMC3278759

[63] Sullivan MJ, Petty NK, Beatson SA. 2011. Easyfig: a genome comparison visualizer. Bioinformatics 27:1009–1010. doi:10.1093/bioinformatics/btr039. 21278367 PMC3065679

[64] Krogh A, Larsson B, von HG, Sonnhammer ELL. 2001. Predicting transmembrane protein topology with a hidden Markov model: application to complete genomes. J Mol Biol 305:567–580. doi:10.1006/jmbi.2000.4315. 11152613

[65] Jones P, Binns D, Chang H-Y, Fraser M, Li W, McAnulla C, McWilliam H, Maslen J, Mitchell A, Nuka G, Pesseat S, Quinn AF, Sangrador-Vegas A, Scheremetjew M, Yong S-Y, Lopez R, Hunter S. 2014. InterProScan 5: genome-scale protein function classification. Bioinformatics 30:1236–1240. doi:10.1093/bioinformatics/btu031. 24451626 PMC3998142

[66] Afgan E, Baker D, Batut B, van den Beek M, Bouvier D, Čech M, Chilton J, Clements D, Coraor N, Grüning BA, Guerler A, Hillman-Jackson J, Hiltemann S, Jalili V, Rasche H, Soranzo N, Goecks J, Taylor J, Nekrutenko A, Blankenberg D. 2018. The Galaxy platform for accessible, reproducible and collaborative biomedical analyses: 2018 update. Nucleic Acids Res 46:W537–W544. doi:10.1093/nar/gky379. 29790989 PMC6030816

[67] Käll L, Krogh A, Sonnhammer ELL. 2004. A combined transmembrane topology and signal peptide prediction method. J Mol Biol 338:1027–1036. doi:10.1016/j.jmb.2004.03.016. 15111065

[68] Project I. Inkscape 1.1. Available from https://inkscape.org.

[69] Schuster-Böckler B, Schultz J, Rahmann S. 2004. HMM Logos for visualization of protein families. BMC Bioinformatics 5:7. doi:10.1186/1471-2105-5-7. 14736340 PMC341448

[70] Wheeler TJ, Clements J, Finn RD. 2014. Skylign: a tool for creating informative, interactive logos representing sequence alignments and profile hidden Markov models. BMC Bioinformatics 15:7–9. doi:10.1186/1471-2105-15-7. 24410852 PMC3893531

[71] Casadaban MJ. 1976. Transposition and fusion of the lac genes to selected promoters in *Escherichia coli* using bacteriophage lambda and Mu. J Mol Biol 104:541–555. doi:10.1016/0022-2836(76)90119-4. 781293

[72] Kolisnychenko V, Plunkett G, Herring CD, Fehér T, Pósfai J, Blattner FR, Pósfai G. 2002. Engineering a reduced *Escherichia coli* genome. Genome Res 12:640–647. doi:10.1101/gr.217202. 11932248 PMC187512

[73] Datsenko KA, Wanner BL. 2000. One-step inactivation of chromosomal genes in *Escherichia coli* K-12 using PCR products. Proc Natl Acad Sci USA 97:6640–6645. doi:10.1073/pnas.120163297. 10829079 PMC18686

[74] Lutz R, Bujard H. 1997. Independent and tight regulation of transcriptional units in *Escherichia coli* via the LacR/O, the TetR/O and AraC/I1-I2 regulatory elements. Nucleic Acids Res 25:1203–1210. doi:10.1093/nar/25.6.1203. 9092630 PMC146584

